# RET mutation heterogeneity in primary advanced medullary thyroid cancers and their metastases

**DOI:** 10.18632/oncotarget.23986

**Published:** 2018-01-04

**Authors:** Cristina Romei, Raffaele Ciampi, Francesca Casella, Alessia Tacito, Liborio Torregrossa, Clara Ugolini, Fulvio Basolo, Gabriele Materazzi, Paolo Vitti, Rossella Elisei

**Affiliations:** ^1^ Endocrine Unit, Department of Clinical and Experimental Medicine, University Hospital of Pisa, Pisa, Italy; ^2^ Department of Surgical, Medical and Molecular Pathology, University Hospital of Pisa, Pisa, Italy

**Keywords:** medullary thyroid carcinoma, RET, genetic instability, tumor clonality

## Abstract

**Purpose:**

Medullary Thyroid Cancer (MTC) whose pathogenesis is strictly related to *RET* proto-oncogene alterations, has been shown to have a heterogenic *RET* mutation profile in subpopulations of MTC. The aim of our study was to investigate the *RET* somatic mutation profile in primary MTC and in the corresponding metastatic tissues in a series of advanced metastatic cases.

**Results:**

This study demonstrated that in about 20% of cases a different *RET* mutation profile can be found when comparing primary tumor and its corresponding metastases. Furthermore in 8% of tumors, *RET* intratumor heterogeneity was observed We also showed that in some cases an imbalance of *RET* copy number was present. We confirmed a high prevalence (90%) of *RET* somatic mutations in advanced tumors.

**Materials and Methods:**

Fifty-six MTC patients (50 somatic and 6 hereditary cases) have been included in the study and a total of 209 specimens have been analysed by direct sequencing. Multiplex ligation-dependent probe amplification (MLPA) has been used to investigate amplification/deletion of *RET* alleles.

**Conclusions:**

In conclusion, this study showed a genetic intra- and intertumor heterogeneity in MTC, But in only 20% of CASES These results could justify the relatively moderate level of aggressiveness of the disease with respect to more aggressive human tumors that are characterized by a high rate of mutation and heterogeneity.

## INTRODUCTION

Medullary thyroid carcinoma (MTC) is a rare endocrine tumor originating from parafollicular C cells of the thyroid. This neoplasia is inherited as an autosomal dominant trait in 25% of patients [1]. In these cases, other organs besides the thyroid (e.g., the parathyroid and adrenal glands) can be involved, thus giving rise to the multiple endocrine neoplasia type 2 (MEN 2) syndromes, which are categorized into three different subtypes (e.g., MEN 2A, MEN 2B and familial medullary thyroid carcinoma or FMTC) according to their phenotype [2]. Activating germline point mutations in the *RET* proto-oncogene have been shown to cause approximately 95–98% of MEN 2 cases [2, 3]. In the other 75% of cases, MTC is a sporadic tumor, and with exception of *RAS* alterations that have been found in approximately 10% of cases [4, 5], somatic mutations in the *RET* proto-oncogene appear to be the most common genetic alteration in MTC tumorigenesis [3]. The most common alterations in the *RET* proto-oncogene are missense gain of function mutations mainly located in the extracellular domain of *RET* (exons 10 or 11) and in the *RET* tyrosine kinase domain (exons 13, 14, 15 and 16).

These mutations are able to cause the constitutive activation of the *ret* onco-protein [6].

In particular, we demonstrated that somatic *RET* mutation prevalence increases with increasing tumor size [7], reaching a prevalence of approximately 90% in advanced cases [8].

The predominant role of a single initiating mutation in MEN 2 was proposed several years ago [9]. Recently, studies performed with deep sequencing technologies, either whole exome sequencing (WES) [10] or targeted sequencing [11–13], have shown that, with a few exceptions, *RET* is the only oncogene altered in MTC.

Over the years, spatial and temporal intratumor heterogeneity have been demonstrated in several human cancers such as clear cell renal cancer, glioblastoma, pancreatic cancer and breast cancer [14–17]. In addition to a different mutation profile, DNA copy number alterations represents an additional feature of intratumor heterogeneity [18]. As far as thyroid cancer genetic heterogeneity is concerned, little is known about the frequency of different mutations in metastases of papillary thyroid histotypes [19–21]. Even less evidence is available on the genetic heterogeneity of MTC. A single study performed on 28 sporadic MTC cases showed an intratumoral heterogenic *RET* mutation profile in 50% of cases [22].

Taking into consideration that *RET* is almost the only oncogene altered in MTC, it is conceivable that, if tumoral heterogeneity exists, different *RET* mutations might be present in different tumoral specimens (i.e., primary and metastatic tissues). The aim of the present study was to investigate the *RET* somatic mutation profiles in a large series of primary MTC cases and corresponding synchronous or metachronous metastatic tissues.

## RESULTS

### RET mutations

Among the 56 metastatic MTC cases included in the study, 7 cases were found to CARRY a *RET* germline mutation (Table 1). However case n. 36 was considered sporadic since it was positive for an A883T mutation in exon 15 that was previously demonstrated, both *in vitro* and *in vivo*, to have a very low or null transforming ability [23, 24]. The remaining 49 patients were found to be negative for the presence of a *RET* germline mutation; thus, a total of 50 cases were considered truly sporadic.

**Table 1:** Available tumoral tissues from the 56 patients with metastatic MTC included in the study

num	Primary tumor	local recurrence (LC)/ metastases	RET germline mutation	num	primary tumor	local recurrence (LC)/ metastases	RET germline mutation
1	n.a.	2 LNF	Neg	29	n.a.	7 liver	Neg
2	1	1 LNF	Neg	30	1	1 LNM	Neg
3	1	1 LC/5 LNF	Neg	31	n.a.	3 LNF	Neg
4	1	1 LNF	Neg	32	1	4 LNF	Neg
5	1	1 LNF	Neg	33	1	1 LNF CC	Neg
6	n.a.	6 LNF	Neg	34	2	2 LNF	C634S
7	1	2 LNF	Neg	35	1	1 LNF	Neg
8	1	2 LNF	Neg	36^*^	3	11 LNF 1	A883T
9	1	1 LNF	Neg	37	n.a.	2 LNF	Neg
10	1	1 LC	Neg	38	1	1 LNF	Neg
11	1	1 LNF	Neg	39	n.a.	3 LNF, 2 brain, 2 liver, 1 kidney, 1 adrenal	C634R
12	1	3 LNF	Neg	40	1	1 LNF	Neg
13	n.a.	2 LNF	Neg	41	n.a.	2 LNF	Neg
14	1	5 LNF, 2 liver	Neg	42	1	1 liver	Neg
15	1	4 LNF	Neg	43	1	1 LNF	Neg
16	1	1 LC/1 LNF	Neg	44	2	1 LNF	Neg
17	1	1 LNF	Neg	45	n.a.	2 LNF	Neg
18	1	1 LNF	Neg	46	n.a.	5 LNF	Neg
19	1	1 LNF	V804M	47	1	2	M918T
20	1	1 LNF	Neg	48	1	1 LNF	Neg
21	1	1 LNF	Neg	49	1	4 LNF, 2 PHEO	M918T
22	1	1 LNF	Neg	50	1	1 trachea	Neg
23	1	12 LNF	Neg	51	1	1 LNF	M918T
24	1	8 LNF	Neg	52	1	1 LNF	Neg
25	1	2 LNF	Neg	53	2	7 LNF	Neg
26	1	2 LNF	Neg	54	1	1 kidney	Neg
27	2	1 LNF	Neg	55	3	3 LNF	Neg
28	2	2 LNF	Neg	56	1	2 LNF	Neg

n.a. not available; ^*^ this patient was considered as sporadic since A883T is not transforming (ref 24) and had the somatic deletion p.D898_E901del, c.2694_2705del12 reported in Table 2. PHEO: pheochromocitoma.

As reported in Table 2, 45/50 (90%) cases were positive for a *RET* somatic mutation, while 5 were *RET* mutation negative. The most frequent *RET* somatic mutation was the M918T mutation in exon 16 that was found in 34/50 (68%) sporadic cases. Other *RET* somatic point mutations or in-frame deletions were found in 5/50 (10%) and 6/50 (12%) cases, respectively. As reported in Table 2, 4/50 (8%) cases were found to carry 2 different somatic mutations, either 2 point mutations or 2 deletions, in the same tissue.

**Table 2:** RET somatic mutations in sporadic cases

Sporadic cases (*n =* 50) RET somatic mutations (45/50 = 90%)	
mutation	n. of cases/50 (%)
M918T^*^	34 (70.4)
C620A	2 (4)
C634G	1 (2)
C634R	1 (2)
A883F	1 (2)
p.D899_E902del, c.2694_2705	2 (4)
p.D898_E901del, c.2692_2703del12	1 (2)
p.Glu632fs c.1894_1904del11	1 (2)
p.Ile638fs c.1912_1918del7^**^	
p.E632_L633del, c.1894_1899del6 + p.D898_E901del, c.2692_2703del12 ^**^	1 (2)
p.E632_L633del, c.1894_1899del6	1 (2)
NM	5 (11.5)

^*^in 2 cases M918T mutation was associated with an additional point mutation in the same tissue (S891A or C620F); ^**^ in 2 cases two different deletions in exon 11 and in both exon 11 and 15, respectively, were found in the same tissue.

### Comparison of RET somatic mutations in different tumoral tissues of the same patient

In the whole series of 56 metastatic MTC cases, we compared the presence of *RET* somatic mutations in different tumoral tissues of the same patient. In the majority of cases (*n* = 45), the comparison was made between primary and metastatic tissues, while in the others (*n* = 11), the *RET* genetic profile was compared in different metastases. The comparison showed that in 45/56 (80.4%) cases, the *RET* mutation profile, either positive or negative, was the same in all the specimens of the same patient. In this group of concordant cases, no double mutants of *RET* were observed. Moreover, in 7 cases (Table 1: n. 27, n. 28, n. 34, n. 36, n. 44, n. 53, and n. 55) of which 2 or 3 different sections of the same primary tissue were available, no differences in the *RET* genetic profile, either positive or negative, were observed.

The other 11/56 (19.6%) cases showed a heterogenic *RET* mutation profile. As shown in Table 3 (panel A), in 5 cases (cases n. 3, n. 18, n. 36, n. 53 and n. 54), a *RET* heterozygous somatic mutation, either point or complex, was present in the primary tumor. This subgroup contained some metastatic lesions that showed the same *RET* mutation as found in the primary tumor, while others were *RET* negative (n. 3, n. 18, n. 36, n. 53). Moreover, case n. 53 showed a lymph node metastases with the same mutation as the primary tumor plus an additional 12 bp deletion of exon 15. Finally, the kidney metastases of case n. 54 that was characterized by a double somatic mutation in the primary tumor showed only one of the two mutations.

**Table 3:** Cases with a different RET mutation profile in different samples

PANEL A			
patient	Primary	LNF	Distant met
3	M918T (1/1)	M918T (2/5) NM (3/5)	n.a.
18	M918T (1/1)	NM (1/1)	n.a.
36	p.D898_E901del, c.2694_2705del12 (3/3)	p.D898_E901del, c.2694_2705del12 (10/11) NM (1/11)	n.a
53	M918T (2/2)	M918T (1/7) p.D898_E901del, c.2694_2705del12 + M918T (1/7) NM (5/7)	n.a
54	M918T/S891A (1/1)	Not present	only M918T (1/1)

PANEL B			
6	n.a.	M918T (5/6) NM (1/6)	n.a.
10	NM (1/1)	n.a.	p.E632_L633del, c.1894_1899del6 p.D898_E901del, c.2692_2703del12 (1/1)
23	NM (1/1)	p.D898_E901del, c.2692_2703del12 (12/12)	n.a.
24	NM (1/1)	C620F+M918T (2/8) M918T (5/8) NM (1/8)	n.a.

PANEL C			
9	M918T homo/hemizygous (1/1)	M918T heterozygous (1/1)	n.a.
14	p.E632_L633del, c.1894_1899del6 heterozygous (1/1)	p.E632_L633del, c.1894_1899del6 heterozygous (4/5) p.E632_L633del, c.1894_1899del6 homo/hemizygous(1/5)	p.E632_L633del, c.1894_1899del6 heterozygous (1/2) p.E632_L633del, c.1894_1899del6 homo/hemizygous (1/2)

n.a. not available

Another subgroup of discordant cases (Table 3, panel B) was characterized by different patterns: a) different metastases of the same patient were either positive or negative for a specific *RET* mutation (n. 6); b) two different *RET* mutations, none of them found in the primary, were present in the same metastatic lesion (n. 10); c) the same *RET* mutation, not found in the primary, was present in all lymphnode metastases (n. 23); d) two different *RET* mutations or just one of them or even none, as it happened in the primary, were found in different metastatic lesions (n 24).

The last two cases (Table 3, panel C) showed a peculiar *RET* genetic profile since in some lesions of the same patient the *RET* mutation was apparently homozygous, while in others it was clearly heterozygous (n. 9 and 14).

In order to verify whether the *RET* negative tumoral tissues belonging to cases with other *RET* positive tissues were false negative due to a low percentage of cancer cells in the sample, we evaluated the prevalence of tumoral cells in the tissue samples. As shown in Table 4, when the percentage of tumoral cells was at least 20% *RET* mutation was detected. Among cases negative for *RET* mutations, some cases had a high percentage of tumoral cells and could be considered real negative instead some cases had low percentage of tumoral cells (10–15%) and they could be potentially false negative. Nevertheless, if we exclude these latter cases, the number of heterogenic cases would not change and affect the final results of the present study.

**Table 4:** Comparison between RET mutation status and percentage of tumoral cells in the analysed tumoral tissue

patient	Type of tissue	% of tumoral cells	RET mutation
3	LNF LNF1 LNF3 primary tumor	n.a. 80 70 20	NM NM NM M918T
53	LNF1 LNF2 LNF3 LNF4 LNF5 LNF6	5 5 20 60 30 n.a.	NM NM p.D898_E901del,c.2694_2705del12 NM NM NM
6	LNF4	20	NM
24	LNF6 LNF7 LNF8 primary tumor	30 10 10 20	M918T/C620F NM NM NM
53	LNF1 LNF2 LNF3 LNF4 LNF5 LNF6 LNF LC SIN	5 5 20 60 30 n.a. 50	NM NM p.D898_E901del,c.2694_2705del12 NM NM NM M918T

### Multiplex ligation-dependent probe amplification (MLPA) assay

**Figure 1:**
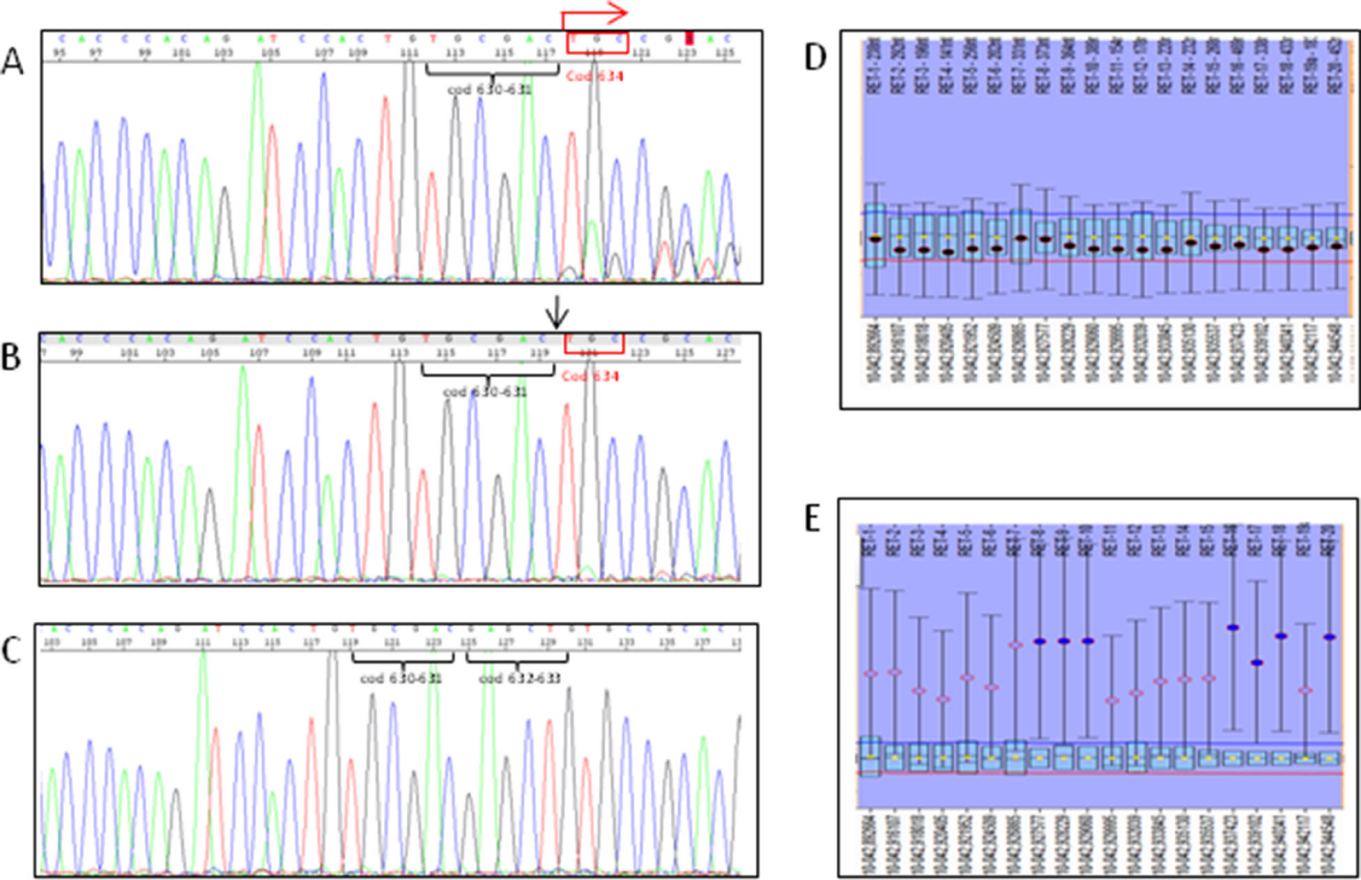
Sanger sequencing pherograms and MLPA graphics of the case n 14 Heterozygous deletion in exon 11 encompassing codons 632–634 (panel **A**) is revealed by the presence of double peaks in the pherogram starting from codon 634 (see red arrow); homo/hemizygous *RET* somatic deletion in exon 11 encompassing codons 632–634 is shown in (panel **B**) and revealed by the absence of both codons 632 and 633 (see black arrow). No *RET* deletion was found at the germline level (panel **C**) as demonstrated by the wild type sequence of *RET* oncogene. MLPA showed that no copy number variation was found within the *RET* gene in the tissues with the heterozygous somatic 6 bp deletion of exon 11 (panel **D**) suggesting a balance between mutated and not mutated alleles. At variance, an amplification of one *RET* allele was observed in the tissue with apparent homozygous 6 bp deletion of exon 11 (panel **E**) suggesting that the amplified allele should be the mutated one.

As previously stated, some tumoral tissues of patient n. 14 showed a heterozygous somatic deletion in exon 11 encompassing codons 632–634 (Figure 1, panel A). Very interestingly, the same deletion was found as homozygous in other metastatic lesions (Figure 1, panel B). No *RET* alterations were found at the germline level (Figure 1, panel C).

MLPA was performed on all tumoral tissues of case n. 14 to determine if the “homozygosity” of the deletion was caused by a *RET* copy number variation. As shown in Figure 1, panel D, no copy number variation was found within the *RET* gene in the tumor that was characterized by the heterozygous somatic 6 bp deletion of exon 11. However, one of the two *RET* alleles was highly amplified with respect to the other in the tissue with the apparent homozygous somatic 6 bp deletion of exon 11 (Figure 1, panel E). Based on these MLPA results, we can reasonably assume that the amplified allele was the mutated one, thus leading to the apparently homozygous pattern observed with the sequencing analysis.

## DISCUSSION

Several studies have demonstrated that *RET* is the most prevalent oncogene involved in MTC tumorigenesis. From the study of the International *RET* consortium [25], the prevalence of *RET* mutations in sporadic MTC cases was found to be approximately 40%, with a high rate of M918T mutations. Over the years, the overall *RET* mutation prevalence was essentially confirmed, but *RET* mutation levels were demonstrated to be significantly lower in small size tumors and much higher in advanced metastatic cases [7, 8, 26, 27]. This latter finding has been confirmed by the results of the present study, since the prevalence of somatic *RET* mutations was 90% in this advanced metastatic MTC series, with M918T mutations being the most frequent. Among the *RET* mutations other than M918T, we found several complex somatic alterations, almost exclusively deletions, affecting exons 11 and/or 15. The presence of these type of *RET* mutations, although more rare than point mutations, has been previously reported in other series, and they have been demonstrated to have high transforming capabilities [28, 29].

Only few cases of sporadic MTC with multiple *RET* mutations in the same tumoral tissue have been reported so far [30]. In our series, we found 4 cases with intratumoral heterogeneity characterized by the presence of 2 *RET* alterations, either point mutations or deletions, in the same tumoral specimen. Although the prevalence of these mixed cases (8% of our series) is rather low, this finding is something new with respect to the results of Eng et al. [22], who found a high prevalence of mixed subpopulations, but only mixed in regards to *RET* positive or negative mutation status. However, it must be noted that the methodology used in the Eng et al study was rather restrictive and did not allow for the identification of all *RET* mutations and in particular could not detect complex mutations. The pathogenic role of a double mutant in the same tissue is still not clear, but Nowell’s evolutionary theory of cancer, which describes how mutations can accumulate in cell subpopulations, remains the most likely explanation [31, 32].

Recently, some studies have described the genetic heterogeneity in different human cancers [14–17]. These findings are rather expected in those human tumors (i.e., lung and melanoma) in which multiple oncogenes (up to 163 and 147 different oncogenes, respectively) have been found to be altered in the same tumor [33–35]. Differentiated thyroid cancers have been shown to have a low number of altered oncogenes, which are usually mutually exclusive [36]. This is particularly true for sporadic MTC, which has a high prevalence of *RET* mutations and a low prevalence of *RAS* mutations, with no other driver oncogenes found so far [3, 5]. For this reason, we concentrated our attention on the *RET* oncogene alone and we showed that 20% of MTC cases were characterized by a different *RET* mutation profile in primary and metastatic tissues. As reported in Table 2, three different patterns of heterogeneity were found, and different hypotheses can be proposed to explain them. The first pattern was characterized by the presence of a *RET* heterozygous somatic mutation in the primary tumor that was not necessarily found in all the corresponding metastases or an additional unique mutation (from that found in the primary tumor) that was found in one metastatic lymph node. To understand this finding, we might hypothesize that the primary tumor is composed of *RET*-positive and *RET*-negative cell subpopulations. Although we cannot be sure that different cells within a tumor can carry different mutations, the study of Eng et al [22] clearly demonstrated that it was possible. As far as the case with the additional mutation is concerned, the most plausible explanation is the evolutionary theory of cancer according to which new mutations can occur in already mutated, developing tumors [31, 32].

In the second pattern, the primary tumor was *RET* negative, and different metastases of the same patient were either positive or negative for a specific *RET* mutation. It is conceivable that a few cells in the primary tumor were positive for the mutations found in the lymphnodes which received a growth advantage during the selective pressures of the metastatic process and produced *RET*-positive metastases [32]. This explanation and the possibility that during the growth of mutated cells other mutations can be added could also justify those cases in which different metastases are genetically different.

The third pattern of heterogeneity showed a peculiar *RET* genetic profile: In some lesions, the *RET* mutation was apparently homozygous, while in others, it was clearly heterozygous. In these cases, a loss of heterozygosity (LOH) (as reported also by Dvorakova et al in their series [30, 37]) or a copy number alteration should be responsible for the observed pattern. The MLPA experiment suggested that an increased number of *RET* mutated alleles were present in our sample. This result agrees with our previous findings, which showed that *RET* gene amplifications were present in *RET-*positive tumors [38].

A limitation of the present study can be due the sensitivity of the Sanger method that has been estimated to be between 10–20% [7, 39, 40]. This implies the possibility to have a technical bias due to the presence of micro metastases, with a low number of metastatic tumoral cells surrounded by normal cells and for these reason not detectable by Sanger sequencing. We tried to rule out this problem and effectively we found that when the tumoral cells were > 20% *RET* mutation was detectable but we also had cases with > 20% of tumoral cells that were *RET* negative. These latter can be considered as true negative cases. For those cases with < 20% of tumoral cells we cannot exclude the possibility they were false negative cases. Nevertheless, the number of heterogenic cases would not change if we exclude these potential false negative cases.

In conclusion, this study showed that a different *RET* mutation profile between primary and metastatic tissues of the same MTC was present in 20% of cases, and in 8% of these, *RET* intratumor heterogeneity was observed. These results, together with the evidence that MTC has a low rate of mutations other than *RET*, explains the relatively moderate level of aggressiveness of the disease with respect to other, more aggressive tumors (lung adenocarcinoma or melanoma) that are characterized by a high rate of mutation and heterogeneity.

## MATERIALS AND METHODS

### Patients

We studied the tumoral tissues of 56 patients (24 females and 32 males) affected with metastatic sporadic or familial MTC. The histological diagnosis and classification of tumoral tissues were performed by an experienced team of local pathologists. MTC cases were included in the study only if at least two tumoral tissues obtained from different lesions were available, either from primary and metastatic tissues or from several metastases. A total of 209 tumoral specimens were analyzed. In particular, 54 primary tumoral tissues, 2 local recurrences, 132 lymph node metastases and 21 distant metastases (i.e., lung, liver, kidney, adrenal gland, brain) were included in the study. The details of the analyzed tumoral tissues and their corresponding patients’ statistics are reported in Table 1.

Tissues were either collected at surgery, immediately frozen in liquid nitrogen and kept at -80°C, or recovered from paraffin-embedded tissue blocks. A peripheral blood sample was available for both sporadic and familial cases.

All patients provided written informed consent. This investigation was approved by our institutional review board and by the local Ethic Committee (protocol number 469, approved 29/1/2015).

### Methods

#### Pathologic diagnosis

Patients underwent total or subtotal thyroidectomy at the Department of Surgery of the University of Pisa, Italy. The presence of typical histological (i.e., tumoral cells arranged in trabecular, insular or sheet-like growth patterns) and immunohistochemical (cells positive for calcitonin and chromogranin) features defined an MTC histological diagnosis. In some cases, the percentage of tumoral cells in the sample was recorded. High levels of serum calcitonin were confirmatory of the C cell origin of the cases.

### Genotyping

DNA was prepared from blood, fresh and paraffin-embedded tumoral tissues according to a previously described protocol [8]. *RET* exons 10, 11, 13, 14, 15 and 16 were analyzed by direct sequencing [41]. To exclude false negative cases, primary tumors and metastases found to be discordant for the presence/absence of the M918T *RET* mutation were further analyzed using a more sensitive TaqMan SNP genotyping assay (ThermoFisher Scientific, Waltham, Massachusetts, USA). The experiments were run according to the manufacturer’s guidelines.

### Multiplex ligation-dependent probe amplification (MLPA) assay

MLPA was used to detect potential large deletions in *RET* gene. Experiments were performed on primary and metastatic tumor samples using the SALSA MLPA P169 HIRSCHSPRUNG PROBEMIX (MRC-Holland, Amsterdam, the Netherlands). Coffalyser.Net software (MRC-Holland, Amsterdam, the Netherlands) was used to identify copy number variations. Experiments were repeated at least twice. Three reference DNAs from the blood of healthy subjects and a negative control (a sample without DNA) were included in all experiments.
